# Integrating systems biology models and biomedical ontologies

**DOI:** 10.1186/1752-0509-5-124

**Published:** 2011-08-11

**Authors:** Robert Hoehndorf, Michel Dumontier, John H Gennari, Sarala Wimalaratne, Bernard de Bono, Daniel L Cook, Georgios V Gkoutos

**Affiliations:** 1 Department of Genetics, University of Cambridge, Downing Street, Cambridge, CB2 3EH, UK; 2 Department of Biology, Carleton University, 1125 Colonel By Drive, Ottawa, K1S 5B6, Canada; 3 School of Computer Science, Carleton University, 1125 Colonel By Drive, Ottawa, K1S 5B6, Canada; 4 Biomedical & Health Informatics, Department of Medical Education and Biomedical Informatics, University of Washington, 1959 NE Pacific Street, Box 357420, Seattle, Washington 98195, USA; 5 European Bioinformatics Institute, Wellcome Trust Genome Campus, Hinxton, Cambridge, CB10 1SD, UK; 6 Department of Physiology & Biophysics, University of Washington, 1705 NE Pacific Street, Box 357290, Seattle, Washington 98195, USA; 7 Department of Biological Structure, University of Washington, 1959 NE Pacific Street, Box 357420, Seattle, Washington 98195, USA

## Abstract

**Background:**

Systems biology is an approach to biology that emphasizes the structure and dynamic behavior of biological systems and the interactions that occur within them. To succeed, systems biology crucially depends on the accessibility and integration of data across domains and levels of granularity. Biomedical ontologies were developed to facilitate such an integration of data and are often used to annotate biosimulation models in systems biology.

**Results:**

We provide a framework to integrate representations of *in silico *systems biology with those of *in vivo *biology as described by biomedical ontologies and demonstrate this framework using the Systems Biology Markup Language. We developed the SBML Harvester software that automatically converts annotated SBML models into OWL and we apply our software to those biosimulation models that are contained in the BioModels Database. We utilize the resulting knowledge base for complex biological queries that can bridge levels of granularity, verify models based on the biological phenomenon they represent and provide a means to establish a basic qualitative layer on which to express the semantics of biosimulation models.

**Conclusions:**

We establish an information flow between biomedical ontologies and biosimulation models and we demonstrate that the integration of annotated biosimulation models and biomedical ontologies enables the verification of models as well as expressive queries. Establishing a bi-directional information flow between systems biology and biomedical ontologies has the potential to enable large-scale analyses of biological systems that span levels of granularity from molecules to organisms.

## Background

Systems biology focuses on the analysis of whole biological systems and interactions occuring within them. For this purpose, it transcends classical boundaries between domains and levels of granularity and follows an integrative approach towards the discovery of biological knowledge. Instead of reducing complex biological phenomena to their basic parts, systems biologists perceive and study these phenomena as components of a network of interrelated processes spanning multiple domains [1]. The methods used to investigate systems' behavior rely on the integration of multi-scale data across levels of granularity, the "integration of *in-silico*, *in-vitro*, and *in-vivo *research" [2], and the construction of computational models to predict and simulate complex systems' behavior. Key challenges that systems biology research faces today are to integrate data within and across domains and levels of granularity, to integrate data that is available in different formats and locations, to provide validation procedures for computational biosimulation models as well as to develop standards for the exchange and integration of models, simulations and results [1-4].

To address these challenges, the research community has proposed standard languages for modelling and model exchange, and has developed common community standards for the annotation of models. In particular, the Systems Biology Markup Language (SBML) [5], the Cell Markup Language (CellML) [6] and the BioPAX standard [7] are standardized model exchange languages that aim to facilitate basic interoperability between the research results that are produced by different research communities. To support the adoption of standard languages, tools that are capable of validating models with respect to their specification language [8-11] or additional guidelines and constraints associated with a modelling standard [12] are available. Model annotation standards include the *Minimum Information Required in the Annotation of Models *(MIRIAM) [13]. MIRIAM specifies which information should be added as metadata to systems biology models and other resources. A part of this metadata is intended to refer to the biological phenomena that a model intends to represent. For this purpose, the annotations can be made using an identifier that belong to an external resources such as a biomedical terminology or ontology. Ontologies focus on a qualitative description of the biomedical domain and are commonly represented in formal languages which facilitate automated discovery of contradictory statements and flexible access to knowledge through automated reasoning.

Most efforts in using ontologies in systems biology modelling are currently limited to the *annotation *of models with ontologies in which the terms from ontologies are treated as *metadata*. We argue that an *integration *of the research areas of systems biology modelling and biomedical ontologies can establish an information flow [14] between them. Such an integration can make a significant contribution towards achieving the aims of system biology while addressing some of the key challenges systems biology faces today. Information flow from biomedical ontologies to systems biology would enable biosimulation modelling and simulation frameworks in systems biology to access the information that is available in data repositories through biomedical ontologies and use this information both to enrich and constrain the scientific analyses of biological systems.

From the perspective of systems biology, the main difference between *annotation *with ontologies and *integration *with ontologies is that annotations are metadata *about *a model, a simulation or a particular result, while integration entails that the ontologies are *used *as a component of a model, a simulation or a result. In particular, when biosimulation models are integrated with ontologies, it becomes possible to translate the structure of a model (within a certain modelling language) into a representation of the biological phenomena that the model represents. An explicit *ontological commitment *[15] of a modelling language is a formal specification of *how *a model's structure corresponds to the structure of the represented phenomena.

Using automated reasoning, the restrictions employed by biomedical ontologies can be applied to verify the *biological consistency *of biosimulation models, i.e., whether or not a model represents phenomena that are biologically possible. Ultimately, integration of computational models and biomedical ontologies, combined with the possibility for model verification, may lead to a genuine standardization of the semantics of modelling languages based on the biology represented through their use: modelling languages would contain a *knowledge-based *layer that formally represents the structure of and the interactions within a biological system.

Here, we demonstrate a method to integrate biosimulation models in systems biology with representations of *in vivo *biology as described by biomedical ontologies in a common formal framework such that information can flow between both disciplines. To evaluate this framework, we apply it to all computational models in the BioModels Database [16] (release 18) that are available in SBML [5] and annotated with biomedical ontologies using the MIRIAM community standard [13]. We provide an ontological analysis of the SBML and specify its ontological commitment [15], i.e., we formalize how the structure of a model in SBML determines the structure of the biological phenomena it represents. We implement the result of our analysis and developed the SBML Harvester software, which converts annotated SBML models into OWL. We use the SBML Harvester to create a formal representation of 269 computational models contained in the BioModels Database. Based on this representation, we verify the consistency of the models and their annotations with respect to the biological phenomena they represent. After establishing the consistency of both, we use the converted models to demonstrate how reasoning over biomedical ontologies can address data integration problems that systems biology faces today: we use the knowledge base of computational models to perform complex biological queries that cross multiple domains and levels of granularity and we demonstrate how to retrieve model elements that represent specific biological phenomena. To achieve these goals, we employ automated reasoning in the expressive description logic underlying the Web Ontology Language (OWL) [17] and automatically infer information from the combined knowledge base that consists of multiple large biomedical ontologies as well as the representation of 269 computational models and their biological semantics.

The SBML Harvester software, a web interface to access the SBML Harvester as well as the ontologies we created are freely available under a BSD license at http://sbmlharvester.googlecode.com.

## Results

### Definitions and scope

The main goal of our proposed framework is the integration between *in silico *biosimulation models and representations of *in vivo *biological phenomena expressed through biomedical ontologies. For this purpose, our method relies on the availability of systems biology resources that are annotated with terms from biomedical ontologies or resources that can be mapped to biomedical ontologies.

We draw a distinction between representations of *in vivo *versus *in silico *phenomena. Representations of *in vivo *phenomena directly represent biological phenomena. Representations of *in silico *phenomena, on the other hand, focus on representing a particular biosimulation model, and biological phenomena are represented only as a secondary consequence, since the biosimulation model is an *in silico *representation of *in vivo *phenomena. In this manuscript, *in vivo *representations also include representations of *in vitro *phenomena, since these methods also deal directly with biological phenomena and molecules.

We use the term *biosimulation model *to describe a mathematical abstraction that is intended to represent the structure, functions, capabilities and qualities of a class of biological systems. A biosimulation model can be expressed in a biosimulation modelling language and executed within a simulation framework. A *biosimulation modelling language *is a formal or semi-formal language that is a component of a biosimulation modelling framework and can be used to specify a biosimulation model. Biosimulation modelling frameworks provide a set of explicit constraints and assumptions about how biosimulation models represent the structure of biological systems and may include guidelines for the documentation and implementation of software systems that support the modelling framework. For example, biosimulation modelling frameworks can fix a specific theory of space and time based on which biological systems are represented within the simulation framework. Simulation frameworks can further define the methods by which connections and transitions in biological systems are performed, or they may include assumptions about the basic types that can be used to represent biological systems. *Types *include both basic data types (such as *Integer *or *Double*) and the classes expressed using conceptual modelling languages (such as *sbml:Model*). Examples of biosimulation models are Guyton's model on circulatory regulation [18] or the model of the cell cycle in yeast [19] contained in the BioModels Database as model BIOMD0000000087. The model BIOMD0000000087 is expressed in the modelling language SBML. Although SBML is primarily intended as a *lingua franca *for the exchange of models and not as a modelling language in itself [5], we will refer to SBML as a biosimulation modelling language within the context of our work. Other biosimulation modelling languages include CellML, MATLAB or BioPAX. Many of these languages evolved into or form part of *biosimulation modelling frameworks *and include guidelines, resources and software tools for the creation, visualization, simulation, documentation and distribution of biosimulation models.

### Model ontology

**Figure 1:**
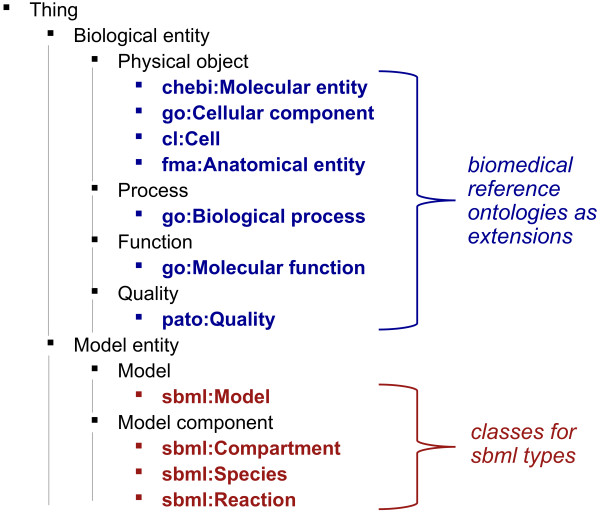
**Taxonomy of a basic upper-level ontology to facilitate integration of in vivo and in silico entities**. Our basic upper-level ontology distinguishes between representation of *in vivo *entities (*Biological entity*) and *in silico *entities (*Model entity*). The sub-classes of *Biological entity *included in the upper-level ontology are *Physical object*, *Process*, *Function *and *Quality*. These classes are further extended by classes from biomedical reference ontologies (e.g., classes from ChEBI, Celltype, FMA, GO and PATO). Sub-classes of *Model entity *include *Model *and *Model component*, and these classes can be extended with SBML-specific classes.

In our method, we employ an upper-level ontology (see Figure 1), both for the *in silico *domain of models and for the domain of biology. Upper-level ontologies provide general classes that are applicable in any domain [20]. The upper-level ontology for biosimulation models is used to facilitate the integration between modelling frameworks and must accommodate the types of entities occurring in these frameworks. The upper-level ontology's role for *in vivo *types is to facilitate the integration of data and knowledge across domains and levels of granularity.

Figure 1 shows our upper-level ontology for both the *in silico *domain of models (on the bottom) and for the domain of *in vivo *biology (on the top). In the first component of the model ontology (bottom part of Figure 1), we include the class *Model entity *and its two subclasses *Model *and *Model component*. To satisfy our SBML-specific use-case, we create an application-specific extension of this ontology that contains the SBML classes *sbml:Model *as subclass of *Model *and *sbml:Species*, *sbml:Compartment *and *sbml:Reaction *as subclasses of *Model component*. These classes are extensions to the upper-level ontology and do not form a part of it. The second part of our ontology (upper part of Figure 1) consists of five classes: the class *Biological entity*, and its four subclasses *Physical object*, *Quality*, *Function *and *Process*. The class *Biological entity *represents particulars, i.e., things that cannot be further instantiated [21]. A process has temporal parts and unfolds through time, while a physical object exists with all its parts at a time point [22]. A quality is an attribute or characteristic of either a physical object or a process [23,24] and we call the potentials or capabilities of physical objects "functions" [25]. The four sub-classes of *Biological entity *are closely interlinked. Physical objects can have qualities and parts whilst functions are determined by an entity's qualities and parts [26]. These functions can then be realized by processes [27]. On each level of distinction in our ontology, the classes are declared as *disjoint*: two classes are disjoint if they cannot have an instance in common.

As an extension to our upper-level ontology, each class in the ontology is further extended with biomedical domain ontologies. We assert the class *Biological process *of the Gene Ontology (GO) [28] as a subclass of *Process*, the *Molecular function *class of the GO as a subclass of *Function*, the *Cellular component *class of the GO as well as all classes from the Celltype Ontology [29], the Chemical Entities of Biological Interest (ChEBI) ontology [30] and the Foundational Model of Anatomy (FMA) [31] as subclasses of *Physical object *and all classes from the PATO ontology [23] as subclasses of *Quality*.

We further introduce and use a set of binary relations, i.e., relations that have two arguments which are filled by instances of classes in our ontology. The **part-of **and **has-part **relations are applicable to both *in silico *and *in vivo *entities. We use the **part-of **relation to assert, as axioms, that model components must be part of a model, that physical objects can only have physical objects as part and that processes can only have other processes as part. The **participates-in **relation relates physical objects to the processes in which they participate and has three sub-relations, based on the different modes of participation distinguished in SBML: **has-input**, **has-output **and **has-modifier**. The **has-input **relation is used to assert that an individual participates in the beginning of a process, **has-output **to assert that an individual participates in the end of a process, while **has-modifier **is used to assert that an individual is essential for a process, participates throughout its occurrence and is not permanently changed by the process [5]. The **function-of **relation is used to assign a function to a physical object and the **realizes **relation can be used to assert that a process realizes a function. The **occurs-in **relation is used to denote the physical object at which a process occurs. Finally, the **quality-of **relation is used to relate a quality to the individual it is a quality of. Here, we assume that a quality is always the quality of exactly one individual. The relations in the model ontology together with associated axioms and inverse relations are listed in table 1.

**Table 1:** List of relations in the model ontology

Relation	Domain	Range	Inverse
part-of	Thing	Thing	has-part
participates-in	Biological entity	Process	has-participant
function-of	Function	Physical object	has-function
realizes	Process	Function	realized-by
occurs-in	Process	Physical object	has-process-occuring
quality-of	Quality	Biological entity	has-quality
input-of	Physical object	Process	has-input
output-of	Physical object	Process	has-output
modifier-of	Physical object	Process	has-modifier

represents	Model entity	Physical object	
model-of	Model	Physical object	

Our main contribution arises from establishing the link between *in silico *model entities and *in vivo *entities in biology. For this purpose, two relations in Table 1 are used to relate model entities to those biological entities they represent. The **represents **relation links model entities, including whole models and model components, to physical objects and the **model-of **relation links models (but not model components) to the physical objects they represent. The use of these two relations allows us to specify *what is being represented *by a computational model and how the components and structure of models in their modelling languages reflect the components and structure of the represented part of the world.

### Formalizing models

Based on the upper-level ontology we developed, we analyze the kinds of entities that are used within a modelling language as well as the kinds of relations in which these entities can stand and explicitly assert what kind of biological entities are represented by a language element (i.e., *how *an element of a modelling language refers to the world). Such an analysis establishes an *explicit correspondence *between the representation of *in silico *methods employed in biosimulation models and the *in vivo *phenomena that they intend to represent and makes the *ontological commitment *[15,32] of a modelling language explicit. To perform such an analysis, classes from several biomedical ontologies can be employed, depending on the modelling framework and the kind of phenomena that the models in this framework represent. To establish the ontological commitment of a modelling language, we assume that models are artifacts of human creation and they attempt to represent possible ways that the world, or a part of the world, could be [33]. The modelling language and its semantics provide constraints and a structure for the description of these worlds, while a particular model characterizes a class of possible biological scenarios. The result of our formalization is a formal characterization of the distinctions that are made by a modelling language as well as how the structure of a model within this language reflects the structure of the biological phenomena it represents.

For the purpose of this analysis, we assume that every model entity represents a *class of physical objects*. For example, a model can be a model of the *Eukaryotic cell *or of entities that have the capability for *Apoptosis*. We assume that model entities that capture the dynamic aspects of a model (such as a *Reaction *in SBML) represent capabilities of physical objects that are realized through processes.

We utilize the MIRIAM compliant annotations of biosimulation models to generate ontology-based representations of these models. In our implementation, we currently restrict our analysis to annotations that use qualifiers that connect a model entity to a class of biological entities using the BioModels.net biology qualifiers *is*, *isVersionOf*, *hasVersion*, *hasPart *and *isPartOf *[34], since these enable us to directly link model entities to existing biomedical ontologies: the qualifier *is *is intended to be used whenever a model entity exactly represents the annotated entity, *isVersionOf *and *hasVersion *are intended to be used whenever the model entity represents a subclass of the annotated entity, *isPartOf *is intended to be used when a model entity is always part of some instance of the annotated entity and *hasPart *is used when a model entity always has some instance of the annotated entity as part. In the Methods section, we present the details about our automatic method for generating OWL representations from the SBML files provided by the BioModels Database and their MIRIAM annotations.

**Figure 2:**
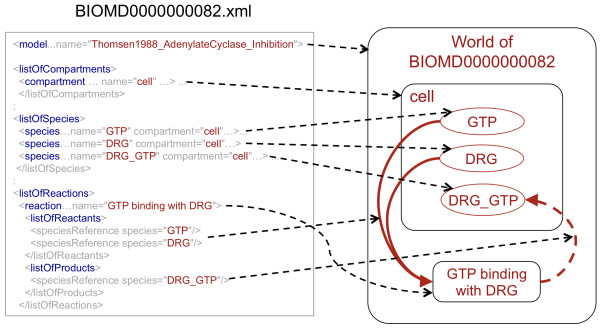
**Schematic representation of a part of the representation generated for **BIOMD0000000082. Here, we demonstrate part of the transformation of the specification of the model BIOMD0000000082 (*in silico*, on the left) into a representation of *in vivo *phenomena (on the right). Each line represents an explicit assertion we create and by which knowledge that is currently implicit in the SBML code is made explicit in an ontology-based, formal representation. The *World of BIOMD0000000082 *has, as a part, a *Cell *(represented by the model's component). The *Cell *has, as its parts, three species (represented by "GTP", "DRG" and "DRG_GTP"). The reaction "GTP binding with DRG" represents a process that **occurs in **the *World of BIOMD0000000082 *and **has **as **input **the objects represented by "GTP" and "DRG", and **has **as **output **the object represented by "DRG_GTP". The input and output relations for processes are inferred from the SBML list of reactants and products, respectively.

As a single example, Figure 2 illustrates parts of the automatically created representation of a specific model of adenylate cyclase inhibition (BIOMD0000000082) within our ontological framework. The left side of Figure 2 shows a simplified and abbreviated snippet of the model's SBML representation. While the model BIOMD0000000082 includes one compartment, six reactions, ten parameters and ten species, we list only one of the model's reactions and three species in Figure 2. The right side of Figure 2 illustrates the representation of the *in vivo *phenomena as produced by the SBML Harvester software. We label the class of entities that BIOMD0000000082 represents "World of BIOMD0000000082". The SBML Harvester software that we described earlier connects the SBML model to the *in vivo *phenomena it intends to represent using the **represents **relation. In Figure 2, assertions of the **represents **relations are illustrated as black dashed lines.

The process of transforming parts of SBML models into OWL makes some aspects of SBML's semantics explicit which are currently implicit in the SBML text. For example, the SBML model BIOMD0000000082 has a single compartment, "cell", which is annotated with the class *Cell *(GO:0005623). We make explicit that this model represents a physical object that *has a cell as part*. Furthermore, species in the SBML model are linked to compartments and we make the assertion explicit that species that are linked to a compartment represent physical objects which are located in the object represented by the compartment. For example, all species in BIOMD0000000082 are linked to the compartment "cell" and therefore all physical objects that these species represent are located in the *Cell *represented by the compartment. We assert that, if a species is annotated with a class *C *using the *is *or *isVersionOf *qualifiers , it represents a kind of *C*. For example, the species "GTP" is annotated (using the *is *qualifier ) with the ChEBI class *GTP *(CHEBI:15996) and therefore we assert that the species labelled "GTP" represents *GTP *that resides in the cell represented by the compartment. In the right side of Figure 2, the nesting of entities represents *parthood *relations.

We assume that reactions in an SBML model are intended to represent processes. In BIOMD0000000082, "GTP binding with DRG" is a reaction that is annotated (using the *isVersionOf *qualifier) with *GTP binding *(GO:0005525). Based on this information, we assert that a *GTP binding *process occurs in *World of *BIOMD0000000082. Furthermore, based on the list of reactants, products and modifiers listed in an SBML model, we assert inputs (using the **has-input **relation), outputs (using the **has-output **relation) and modifiers (using the **has-modifier **relation) to the process. For example, the species "GTP" and "DRG" are listed as reactants of "GTP binding with DRG". Based on this information, we assert that the reaction "GTP binding with DRG" represents a *GTP binding *process that has two entities as input: the entity represented by the species "GTP" and the entity represented by the species "DRG". Similarly, we formalize the list of products of "GTP binding with DRG" and assert that the reaction represents a *GTP binding *that has a physical object as output which is represented by the species "DRG_GTP". In Figure 2, inputs of reactions are illustrated with red lines and outputs are illustrated with dashed red lines. The Methods section describes the precise method for formalizing SBML models.

We developed the SBML Harvester software that automatically converts SBML models into OWL using this method and our basic model ontology. The Harvester creates an integrated representation of both the structure of a model and the biological phenomena that a model intends to represents. We applied this software to the BioModels Database, release 18, and automatically converted 269 models. The result of this conversion is a knowledge base (which we will refer to as *BioModels knowledge base *within this manuscript) composed of more than 90,000 classes which are characterized based on more than 800,000 logical axioms (i.e., axioms restricting either classes or object properties). The knowledge base includes the GO [28], the ChEBI Ontology [30], the Celltype Ontology [29], the Foundational Model of Anatomy [31] and the PATO Ontology [23], all of which are freely available from the OBO Foundry ontology repository [35]. In addition to these ontologies, we create two classes for each model, compartment, species or reaction, one for the *in silico *entity represented within SBML, the other for the *in vivo *entity characterized through biomedical ontologies. Furthermore, in some cases, a class of concentrations, charges or amounts is created for species. The details of the conversion process are described in the Methods section. The knowledge base and the software to convert SBML models are available as supplementary material on our project website.

### Consistency verification

Automated reasoning over the BioModels knowledge base provides a means to verify models and their annotations. Upon applying this analysis to the formalized BioModels Database, we detect several contradictions in the knowledge base. These contradictions arise from annotations in 27 models. For example, in a model of the mitotic cell cycle (BIOMD0000000056) we identify 6 annotations as problematic: the model contains a compartment annotated with *Cell*, in which the species "SPN" annotated with *spindle assembly *(GO:0051225) is located. According to our analysis, however, species represent physical objects, while *Spindle assembly *is a kind of process. Consequently, the annotation of a species with a process class will cause an inconsistency that we automatically detect. Another type of consistency violation in the same model relates to the annotation of a reaction which is annotated with a class of physical objects. The reaction "Assoc with NET1P to form RENTP" is annotated with *RENT complex*, a subclass of cellular components. However, according to the method we propose, a reaction may represent something that has a RENT complex as participant, but it cannot represent a kind (i.e., a subclass) of physical object.

### Kinds of consistency violations

#### Mixing physical objects, functions and processes

The most basic problem that leads to a contradictory class definition is the disjointness of physical objects, functions and processes. We assume in our analysis that species and compartments represent physical objects, while reactions represent functions or processes. However, in some cases, a species is annotated with a function or process with the intended meaning that the species is a physical object that either has the annotated function, or a species that has some function that is realized through the annotated process. For example, in BIOMD0000000008, the species "protease" is annotated with *Peptidase activity *(GO:0008233) with the intention that the species represents *Peptidase*, i.e., molecules with the function *Peptidase activity*.

In most cases, an automated reasoner is capable of disambiguating the intended meaning. For models in the SBML, the following three interpretations are disjoint:

• the model is a subclass of *C*,

• the model is a subclass of things that have the function *C*, and

• the model is a subclass of things that have a function that must be realized by *C*.

In each of these alternatives, *C *is interpreted differently: as a kind of physical object, a kind of function or a kind of process. Because all these interpretations of *C *are disjoint, an automated reasoner will eliminate two options and select exactly one [36].

A similar list of possible interpretations of annotations can be created for SBML's reactions. Although the incorporation of these disjunctive interpretations of annotations would lead to a formally consistent knowledge base, incorrectly annotated model entities would not cause an inconsistency anymore and therefore prevent us from automatically identifying incorrect annotations through automated reasoning. In our SBML use case, we perform a disjunctive interpretation for annotations to *Model*, but not for annotations to *Species*, *Compartment *or *Reaction*.

#### Extending features of modelling languages

In some cases, inconsistencies in the knowledge base indicate the use of modelling language features for purposes different from what may have been intended by the language designers. For example, in BIOMD0000000087, SBML's species elements are used to represent temporal stages. The model BIOMD0000000087 is a model of the cell cycle in yeast [19] and includes a compartment annotated with *Nucleus *that contains, among others, the species *G1*, *G2*, *S *and *M *annotated with *G1 phase*, *G2 phase*, *S phase *and *M phase*, respectively. Within the model, the amounts of these species can be either 0 or 1, and the initial amount of *G1 *is 1 and the initial amounts of *G2*, *S *and *M *are 0. The values of these species serve as a basic representation of temporal stages of the cell cycle, and the species' values are used as preconditions for reactions within the model. While the choice to represent stages of the cell cycle as species will not lead to biologically incorrect models and may be motivated by limitations in the simulation environment, our method can be used to identify the cases in which the intended use of SBML by modellers does not coincide with the ontological representation of SBML features. With an appropriate extension of the biology qualifiers that can be used to link model entities to biological entities, we would be able to incorporate such model annotations.

#### Biological impossibility

A third kind of contradictory class definition that we can automatically identify is due to a violation of biological constraints. For example, recently added definitions of classes in the GO [37] define an *ATPase activity *(GO:0004002) as a *Catalytic activity *that has *Water *and *ATP *as input, *ADP *and *phosphate *as output and is a part of an *ATP catabolic process*. *Water *and *ATP *are classes in the ChEBI ontology that cannot have common instances and therefore should be declared as disjoint classes, although such a restriction is not yet included in ChEBI. Similarly, although the GO class definitions do not explicitly state that *ATP *and *Water *are *the only *inputs of an *ATPase activity*, such a restriction may be added to the GO. To demonstrate the utility of ontologies for identifying biologically inconsistent classes, we add the assertion that *Water*, *ATP *and *Alpha-D-glucose 6-phosphate *are disjoint as well as the definition of *ATPase activity *to the knowledge base that we generated.

When querying for models containing contradictory definitions in this extended knowledge base, we obtain BIOMD0000000176 and BIOMD0000000177 as a result. The models BIOMD0000000176 and BIOMD0000000177 are models of anaerobic glycolysis in yeast [38] and both contain a species labelled "ATP" that is the input of a reaction labelled "ATPase". The reaction "ATPase" is annotated with *ATPase activity*. The species "ATP", however, is annotated with *Alpha-D-glucose 6-phosphate *(CHEBI:17665), not with *ATP*. Based on the definition of *ATPase *in the GO (and the additional assertions we add to the knowledge base), the class that is represented by the *ATPase *reaction becomes unsatisfiable, and this problem is automatically identified through reasoning. In the two models, the cause of the inconsistency is an incorrect annotation of the species "ATP" with *Alpha-D-glucose 6-phosphate *instead of *ATP*. As ontologies are further extended with expressive class definitions [37,39,40], more of these annotation problems can automatically be identified and subsequently corrected.

A consistent representation of knowledge is necessary to utilize automated reasoning. The correction of the problems that we identified in the annotations and models contained in the BioModels Database requires substantial knowledge about both the model structure and their applications. Therefore, we do not perform a manual repair of the identified inconsistencies and ignore the 27 models that contain contradictory class definitions in further analyses.

### Knowledge discovery and composition

#### Retrieving models

We utilize the resulting knowledge base for automated reasoning to perform inferences across the combination of models and the biological phenomena they intend to represent. Since these phenomena are described using biomedical reference ontologies, inferences can take the information contained in these reference ontologies into account to perform queries across multiple connected domains as well as distributed knowledge bases. Table 2 shows a list of queries for the BioModels knowledge base, including the number of results. We obtained these results using the Pellet OWL reasoner [41] and the Protege ontology editor [42].

**Table 2:** List of examples for querying the BioModels knowledge base

Query	Query string	# results
Contradictory defined entities	Nothing	4,899

Models which represent a process involving sugar	model-of some (has-part some (has-function some (realized-by only (has-participant some sugar))))	54

Parts of BIOMD0000000015 that represent processes involving sugar	part-of some BIOMD0000000015 and represents some (has-function some (realized-by only (has-participant some sugar)))	29

Model entities that represent the cell cycle	represents some (has-part some (has-function some (realized-by only 'cell cycle')))	14

Model entities that represent mutagenic central nervous system drugs in the gastrointestinal systems	represents some (has-part some ('has role' some 'central nervous system drug' and 'has role' some mutagen and part-of some 'Gastrointestinal system')	2

Model entities that represent catalytic activity involving sugar in the endocrine pancreas	represents some (has-function some (realized-by only (realizes some 'catalytic activity' and has-participant some (sugar and contained-in some (part-of some 'Endocrine pancreas')))))	4

List of examples for querying the BioModels (release 18) knowledge base. The results of these queries are based on automated reasoning (i.e., deductive inference). Every result listed in the table is the result of a formal proof that is based on the constraints formalized in the SBML Harvester software, the annotation assertions of models in BioModels and the knowledge contained in biomedical ontologies.

If an answer to any of the queries is incorrect, then this inaccuracy must be the consequence of either an incorrect assertion in a biomedical ontology, an inappropriate use of SBML model elements, an incorrect model, a faulty model annotation or a mistake in our assumptions about SBML's ontological commitment.

Quantifying these cases and correcting the underlying problems requires a manual analysis of the models and their annotations, and we intend to collaborate with the BioModels Database curators on identifying and correcting possibly incorrect model annotations. The SBML Harvester software can further provide the means to verify models before inclusion in the BioModels Database.

Using inference over both ontologies and the formalized model structures, we are able to ask detailed queries, most of which are currently not possible with the current BioModels Database query interface. In addition, use of automated reasoning enables us to utilize the biological semantics of models explicitly, in contrast to search methods based on lexical matching that rely on names and terminology.

The most basic question we can ask is the retrieval of models which represent an entity of a certain kind. For example, we can query for models (or parts of models) that represent the *Cell cycle *and, based on the ontology-based annotation of models and our conversion, we retrieve 14 models.

Via the integration of biomedical ontologies into our framework, we can also perform queries that rely on the ontologies' axioms. For example, we are able to query for models that represent biological processes in which *sugar *(CHEBI:16646) participates. This query depends on the ChEBI ontology [30], the Gene Ontology (GO) [43] as well as the complex ontology-based descriptions that result from the application of our method. As a result of performing this query over the ontological representation of the BioModels Database, we obtain 54 models (e.g., BIOMD0000000015, a model of purine metabolism).

#### Retrieving model components

We can further refine our query to retrieve all parts of this model that represent processes involving sugar and retrieve 29 reactions. For example, the reaction labelled "pyr" has 5-O-phosphono-alpha-D-ribofuranosyl diphosphate (CHEBI:17111) as input, which is a kind of *sugar*. We can extend this query further: we can add restrictions taken from reference ontologies (e.g., query for sugars that have a phosphorus atom as part; sugars that have an oxoacid as functional parent) or add restrictions arising from the model (e.g., sugars located in a cell; sugars that are the input of biosynthetic processes that have ATP as output).

#### Bridging levels of granularity

Combining the inferences over the biomedical ontologies and the representation of computational models enables the specification of queries of high biological complexity as well as using automated reasoning over biomedical ontologies to bridge levels of granularity. For example, we can query our ontology for any model that represents the uptake of any mutagenic central nervous system drug by the gastrointestinal system. Using the terms *Mutagen *(CHEBI:25435) and *Central nervous system drug *(CHEBI:35470) from the CHEBI ontology as well as *Gastrointestinal system *(FMA:71132) from the FMA enables us to retrieve a model of the tolerance to pressor effects in caffeine uptake (BIOMD0000000241).

Furthermore, we can use ontologies to perform queries for anatomical or physiological phenomena and retrieve specific molecular biosimulation models that satisfy the specified conditions. For example, we can query the BioModels knowledge base for models that represent catalytic activities involving sugar in the endocrine pancreas and obtain a model of phosphofructokinase and glycolytic oscillations in the pancreatic beta-cell [44] (BIOMD0000000236) as result. This model has a compartment annotated with *Type B cell of pancreatic islet *(FMA:70586), and according to the FMA, these cells are part of the *Pancreatic islet *which is part of the *Endocrine pancreas*. Furthermore, this compartment contains several species annotated with subclasses of *Sugar *(e.g., *D-fructose 6-phosphate*) which are inputs and outputs of reactions that represent catalytic activities (e.g., *6-phosphofructokinase activity*).

Since we have combined multiple models in a single ontology, we can ask for connections between models based on connections between the biological phenomena they represent. This has the potential to lead to a powerful ontology-based method for composing models. For example, we can query our ontology-based framework for kinds of processes that are part of the cell cycle and then ask for models or model components that represent them. Based on these components, we could then construct a new model that consists of the components necessary to represent the cell cycle. A method for automatically or semi-automatically generating models based on ontology-based queries is a viable area for future research.

### Performance of reasoning

To apply our method within software systems such as simulators, or utilize it as part of an analysis method, it is critical that queries be answered efficiently, especially when a large number of queries are considered. Our method uses automated reasoning in OWL, and, in the worst case, the time needed to answer queries increases exponentially with the number of logical axioms in the ontology [45]. Using the Pellet OWL reasoner [41] on hardware consisting of two Intel^® ^Xeon^® ^2.4 GHz quad-core CPUs with 24 GB memory, classification of the BioModels knowledge base requires more than one hour while the time required to answer queries depends on the query's complexity and ranges between 1 and 30 minutes in our experiments.

To address this problem, we leverage recent research around the development of *OWL profiles *[46] that restrict OWL's expressivity in order to improve the speed of automated reasoning and, in particular, to guarantee polynomial-time reasoning. To utilize reasoning in these profiles, we use the EL Vira software [47] to perform an automatic conversion of the BioModels knowledge base into the OWL EL [46,48] profile and then applied the CEL reasoner [49]. Using CEL after applying the EL Vira modularization approach, we were able to classify the BioModels knowledge base in less than one second and perform queries in 3 to 10 milliseconds.

## Discussion

### Limitations and related work

#### Ontologies in systems biology

The potential to utilize biomedical ontologies for the discovery and integration of models has been recognized within the systems biology community, and several efforts make use of ontologies. Both domain-specific ontologies that target a particular aspect of systems biology are being developed and applied, and ontologies for several areas of biology are used to annotate, retrieve and compose biosimulation models. For example, the Systems Biology Ontology (SBO) [50] is applied, amongst others, within the SBML [5] to describe and restrict model components. The Model Format Ontology (MFO) [12,51] further provides structural restrictions and constraints for the SBML and the BioPAX standard [7] is based on an ontology for representing pathways and reactions. The KiSAO and TEDDY [52] domain ontologies can be used to characterize the dynamic aspects of systems' behavior. Model repositories, including the BioModels Database [16], contain large corpora of models, some of which are richly annotated with classes from biomedical ontologies.

Annotations of biosimulation models with biomedical domain ontologies are already widely being applied for knowledge extraction and discovery [53-55] as well as model composition [56-60]. Statistical methods and semantic similarity [61] can be used to rank models, identify similarity between model components and suggest modules that can be recombined into new models. Methods based on semantic similarity provide a powerful means to identify and discover associations between entities that are annotated with ontologies and rank them based on how similar two entities are with respect to a certain similarity metric. However, semantic similarity methods use the intended meaning of ontology-based annotations only *indirectly *and often rely on distance measures between nodes in graphs, information content or set comparisons [62]. Our method, on the other hand, uses the intended meaning of model annotations *directly *through deductive inference. The use of deductive inference guarantees *absolute certainty *about inferred statements [63], provided that the biomedical ontologies and model annotations that were used for the inference are correct. For example, all results to queries that we list in Table 2 are the result of a formal proof across the combination of biomedical ontologies and the formalized models in our knowledge base.

Automated reasoning can reveal complex relations between both model entities and terms in biomedical ontologies, while similarity-based approaches have the potential to suggest genuinely novel hypotheses that cannot be derived from existing knowledge. Consequently, our method can complement existing knowledge extraction and knowledge discovery methods in systems biology: first, ontology-based annotations are verified, formalized and additional knowledge inferred through automated inference, as described in our method; in a second step, established methods for knowledge discovery, ranking and model composition can be utilized based on the verified and enriched representation generated through our method.

Similarly to our method, the Rule-Based semantic Mediation (RBM) method [51] has been used to apply automated reasoning and enrich the knowledge available within a biosimulation model. To achieve this goal, RBM uses the MFO [12] to represent the syntax of SBML in OWL, embeds several biological databases in a common ontology-based model, adds an expressive core ontology about the biological domain that is being modelled and interrelates syntactic information and knowledge contained in the core ontology using rules. Through the combination of these methods, RBM can partially automate the model annotation process and it has been demonstrated that RBM is able to infer novel and biologically meaningful relations between the entities that are represented within a model [51]. In contrast to our method, RBM does not formalize and implement the ontological commitment of the modelling language itself nor does it provide a foundation of model entities in an upper-level ontology that integrates *in silico *and *in vivo *entities. Furthermore, RBM does not yet utilize the large number of pre-existing biomedical ontologies for inferences. Instead, RBM relies on the creation of a core ontology containing expressive axioms to capture the constraints in the biological domain. We make use of biomedical ontologies that rarely use expressive axioms and their weak formalization limits the capabilities of our method for verification and knowledge extraction. Therefore, a combination of our approach with RBM is a viable subject for future research.

Further related work on data integration in systems biology and biosimulation includes the work on BioPAX [7] and its integration with SBML [64] as well as the integration of SBML with the Taverna workflow system [65]. While we have not used these approaches to improve our framework yet, we plan to combine our framework with different methods for data integration in systems biology in the future.

#### Availability of model annotation

A bottleneck in our method is the availability of ontology-based annotations for systems biology resources. While our method can be used to generate complex definitions from these annotations and verify them with respect to biomedical ontologies, it relies on the availability of ontology-based annotations. To annotate models, software tools such as semanticSBML [56], Saint [66], Metannogen [67] and other MIRIAM-aware modelling tools [68-70] and annotation libraries [71] will continue to play a prominent role. The software we developed could be added as a component to existing model annotation frameworks to verify the annotations which are added by curators, and we make our source code available for this purpose.

#### Complexity and expressivity of automated reasoning

Another limitation of our work is the complexity of reasoning over ontologies in OWL. To address this problem, we have used a method of converting the ontology created by the SBML Harvester to OWL EL, a fragment of OWL that enables polynomial-time automated reasoning. However, OWL EL does not support the use of the universal quantifier ("only"), negation or inverse relations, and queries that involve these OWL constructs can no longer be performed. In our conversion, we make use of the universal quantifier to relate functions to the processes that can realize them. The use of universal quantification is required to ensure that functions can remain unrealized [39,72] (i.e., there is not always a process that realizes a function). As a consequence, we can no longer answer queries for the phenomena represented by SBML's *Reaction *model elements since we use universal quantification in their formalization (see Methods section) and these axioms are lost after conversion to OWL EL.

However, although the use of the universal quantifier is applied in several biomedical ontologies [73,74] and applying it in our framework will improve interoperability with these ontologies, we may also replace the universal quantification with another construct (involving, for example, existential quantification) to enable more expressive queries at the cost of interoperability with some ontologies. For this purpose, we implemented an option in the SBML Harvester software to create OWL EL ontologies as output. When this option is activated, the universal quantifier is replaced with an existential quantifier in the formalization of process- and function-based annotations and we import only ontologies (including our upper-level ontology) that we converted into the OWL EL fragment using the EL Vira software [47]. Investigating alternative formalizations of the constraints employed in the SBML Harvester to improve query performance and expressiveness is an important subject for future research in collaboration with the users of our system.

#### Upper-level ontologies

We utilize a basic upper-level ontology, as shown in Figure 1, and we intend to expand this ontology in the future. In the case of SBML, the MFO [12] represents the structural constraints of the modelling language and can be used to extend the *in silico *entities within our upper-level ontology (lower part of Figure 1). Established ontologies such as the General Formal Ontology (GFO) [21,22], the Descriptive Ontology for Linguistic and Cognitive Engineering (DOLCE) [75], the Suggested Upper Merged Ontology (SUMO) [76] or the Basic Formal Ontology (BFO) [77] are comprehensive upper-level ontologies and can replace the *in vivo *side in our upper-level ontology. While established upper-level ontologies can provide more classes for *in vivo *entities as well as useful axioms and distinctions [20] that could improve our method further, they can also enforce a philosophical commitment that is not necessarily shared across domains and communities [78-81]. Therefore, we utilize a minimal upper-level ontology that specifically addresses our use-case and which can be mapped to existing upper-level ontologies if and when needed. In the upper-level ontology we employ, we only commit to those distinctions that are necessary for implementing and demonstrating our method. In the future, we intend to collaborate with other researchers, the developers of tools for processing model annotations and the users of our system to provide a more comprehensive framework that can accurately represent all the types of entities that are used within specific modelling frameworks.

### An ontology-based layer for model exchange and verification

While it is uncertain whether a single standard representation and exchange language for systems biology models will ever be agreed upon, there is a great potential for unifying the *semantics *of these languages on their *ontological level*. In other words, a model of *Apoptosis *can be represented using SBML, CellML, MatLab, Fortran, and several other languages, and agreeing on a single common language that is suitable for all purposes and simulators is as unlikely as agreeing on a single programming language. However, agreeing on the meaning of "Apoptosis" and formally specifying this meaning using a class *Apoptosis *seems much more achievable, in particular as *Apoptosis *is already specified and defined in ontologies such as the GO. Consequently, while the syntax of model representations may never be combined in a single uniform format, their underlying *ontological commitment *may provide a means for achieving genuine interoperability and integration between models and modelling languages. Annotation of computational models alone will not achieve this goal, since genuine integration requires a framework for information flow between the structure of the model and the biological phenomena it represents. Despite considerable efforts in semantically representing and characterizing models [50,56-58], such an integration has not yet been achieved. The framework we propose demonstrates a strategy for integrating parts of systems biology and biomedical ontology, and we believe it has the potential to address some of the key challenges that systems biology faces today.

Identifying only 27 models that violate the formal constraints we place on the use of SBML and its annotations demonstrates the great care that the BioModels Database curators have used in their model annotation efforts. Based on this well-curated database, we could demonstrate that our framework has the potential to facilitate the verification of the biological consistency of model annotations through automated reasoning. Since model annotations are currently used as *metadata*, a verification of the models based on their annotations is not yet common. Consequently, the majority of the contradictions we identify arise from the model annotations and not the models *per se*. However, if the model annotations would cease to be treated as metadata and become part of a standard according to which models can be verified, our method could be of utility to curators in their annotation task as well as aid in the discovery of modelling problems.

We have identified three major kinds of inconsistencies in the BioModels Database. The first group of inconsistencies is due to our ontological analysis of SBML and the resulting restrictions we place on the use of its components. In particular, while we assume that some language elements in SBML refer exclusively to physical objects, functions or processes, it is possible to refer to physical objects through their function (e.g., objects with the function to secrete insulin), or to functions through the processes that occur when the function is realized (e.g., functions that are realized through ATP binding processes). Although we show how our analysis could be extended to accommodate these three alternative interpretations in each case, such an extension would hide other problems in the BioModels knowledge base from automated discovery. Instead, an appropriate extension and use of annotation qualifiers [13,34] could help to provide a consistent and verifiable transformation of annotated SBML models into OWL.

A second group of inconsistencies arises through the use of SBML language features for purposes different from what they are commonly used for. In particular, we have identified a model in which SBML's *species *are used to simulate a basic theory of . In this model, species represent temporal intervals and are consequently annotated with *Process *terms. The underlying ontological commitment of SBML's use in such a model is substantially different from the constraints that we make explicit through our analysis, and consequently, we cannot consistently incorporate this model in our knowledge base. To accommodate these models, the modelling language could be extended to include additional features when not available (such as an explicit representation of temporal stages that can be used to constrain reactions within a model) or additional model annotation qualifiers can make such complex relations between model elements and their intended meaning explicit.

Finally, the third group of consistency violations is due to constraints in biomedical ontologies that further restrict biosimulation models and their annotations. Consistency violations of this group arise when a model represents biological phenomena that are *impossible *according to the current knowledge of biology, as expressed in biomedical ontologies. To detect problems of the third kind, the first two groups of problems must be resolved and expressive, richly formalized ontologies must be used.

### Extension to other modelling languages and domains

To apply our framework across modelling languages, the part of our model ontology that represents *in silico *entities must be extended to accommodate the kinds of model entities that are used in other modelling languages. In addition, the ontological commitment of such modelling languages must be made explicit. For example, to apply our method to CellML, our model ontology must be extended with CellML specific types. Based on such an extended ontology, the correspondence between the structure of CellML models and the structure of the biological phenomena that they represent must be established. As a result, models in different modelling languages can be compared based on an ontological characterization of the biological phenomena they represent.

To achieve this goal rapidly, conversion methods between modelling languages can be used, such as a conversion between CellML and SBML [82]. The CellML2SBML effort establishes a mapping from CellML language elements to identifiers, units, compartments, species, parameters, reactions and rules in SBML, and consequently, our SBML conversion could be used to provide a representation of models converted through this method. However, both for the tasks of consistency verification and data retrieval, a CellML-specific ontological analysis would be preferable to the use of such a translation, since some features of CellML may be lost through a conversion into another modelling language.

A further extension of our method is to incorporate the dynamic aspects of modelling. Models, according to our analysis, represent static entities in which only the capabilities for processes are present.

Simulations of models, on the other hand, represent processes in which the entities represented by the model components participate. For this purpose, a simulator can trigger the functions of model entities that represent physical objects, and, as future work, the complex relations between biosimulation models, the simulations and the structures and processes that both represent can be made explicit. For this purpose, our method could be extended to accommodate the Minimum Information About a Simulation Experiment (MIASE) [83] standard and could apply a similar approach as we demonstrated here with SBML to the SED-ML language [84]. The extension of our method to incorporate biosimulations and their results is subject to future research.

## Conclusions

We have demonstrated how to formalize the biological meaning of models in systems biology. We used this formalization to both validate and verify the biological consistency of models, as well as to demonstrate semantic retrieval of biosimulation models based on the structure of the biological phenomena they represent. Together, these capabilities have the potential to improve access and understanding of models, and ultimately to integrate biosimulation knowledge across domains and levels of granularity.

For large-scale analyses and applications, we demonstrate that a reduction into a tractable fragment of OWL (OWL EL) is required so that inferences can be performed efficiently. Our method is applicable to any kind of information that can be represented using SBML and annotated with biomedical entities using the BioModels.net biology qualifiers. We have applied our method to the BioModels Database as well as to YeastNet, a consensus metabolic network for yeast [85], and make the resulting OWL ontologies as well as the SBML Harvester software we created freely available on our website.

While ontologies in biomedicine have traditionally been limited to single domains [35], recent achievements contributed to making these ontologies interoperable by characterizing their relations [74,86], specifying the semantics of ontology representation languages used for biomedical ontologies [87,88] and incorporating axioms that link the classes of one ontology to classes in other ontologies [37,40,89]. The links between the classes of multiple ontologies provide relations that facilitate data integration across domains, levels of granularity and species. Similar dimensions of data integration are faced by systems biology. Therefore, an integration of biomedical ontologies and systems biology models can provide a formal representation framework based on which the data relevant to the study of biological systems can be integrated across domains, granularity and species, and made available to scientific analyses of biological systems.

## Methods

### Formal ontology, ontological commitment and the axiomatic deductive method

An ontology is the specification of a conceptualization of a domain and is used to make the meaning of terms in a vocabulary explicit [15]. A conceptualization is a system of categories accounting for a particular view on the world [15]. The relation between a vocabulary and a conceptualization is the *ontological commitment *of a language: the ontological commitment assigns a category from the conceptualization to each term in a vocabulary. Therefore, the ontological commitment of a language specifies the *meaning *of terms in a vocabulary, i.e., it determines *how *a term refers to the world and *what *kind of phenomenon it represents.

A specification of a conceptualization in a formal language follows the axiomatic-deductive method [63,90]. A term *t *in a vocabulary can be *defined *through an explicit definition : a definition of *t *is a statement in which *t *does not appear and which can be substituted for every occurrence of *t *in other statements. Terms that occur in *t*'s definition must again be defined based on other terms. Eventually, a set of *primitive *terms remains which are not defined further. Instead, the primitive terms are characterized through a set of sentences which are assumed to be true in the investigated domain, and these sentences constitute the *axioms *of the ontology. Ideally, the axioms are chosen in such a way that, through the use of deductive inference, all sentences that are true in the investigated domain can be inferred from the axioms [90].

### SBML conversion

In the conversion method applied by the SBML Harvester, every element *E *of the SBML language represents a class *Rep*(*E*) and we assert that E SubClassOf: represents some Rep(E). We use the following rules to restrict *Rep*(*E*) further:

• If a model *M *is annotated (using the BioModels.net biology qualifiers is, isVersionOf or hasVersion) with the physical object classes *O*_1_,..., *O_n_*, we assert: Rep(M) SubClassOf: has-part some*O*_1 _and ... and has-part some*O_n_*.

• If a model *M *is annotated (using the BioModels.net biology qualifiers is, isVersionOf or hasVersion) with the function classes *F*_1_,..., *F_n_*, we assert: Rep(M) SubClassOf: has-part some (has-function some*F*_1_) and ... and has-part some (has-function some*F_n_*).

• If a model *M *is annotated (using the BioModels.net biology qualifiers is, isVersionOf or hasVersion) with the process classes *P*_1_, ..., *P_n_*, we assert: Rep(M) SubClassOf: has-part some (has-function some (realized-by only*P*_1_)) and ... and has-part some (has-function some (realized-by only*P_n_*)).

• If a compartment *C *in a model *M *is annotated (using the BioModels.net biology qualifiers is, isVersionOf or hasVersion) with the physical object classes *O*_1_, ..., *O_n_*, we assert: Rep(C) SubClassOf:*O*_1 _and ... and*O_n _*and part-of some Rep(M).

• If a species *S *in a compartment *C *is annotated (using the BioModels.net biology qualifiers is, isVersionOf or hasVersion) with the physical object classes *O*_1_, ..., *O_n_*, we assert: Rep(S) SubClassOf:*O*_1 _and ... and*O_n _*and part-of some Rep(C).

- If *S *has an initial concentration, we further create the restriction: Rep(S) SubClassOf: has-quality some PATO:0000033, where *PATO:0000033 *is the quality labelled *Concentration*.

- If *S *has an initial charge set, we create the restriction: Rep(S) SubClassOf: has-quality some PATO:0002193, where *PATO:0002193 *is the quality labelled *Charge*.

- If *S *has an initial amount set, we create the restriction: Rep(S) SubClassOf: has-quality some PATO:0000125, where *PATO:0002193 *is the quality labelled *Mass*.

• If a reaction *R *in a model *M *is annotated (using the BioModels.net biology qualifiers is, isVersionOf or hasVersion) with the function classes *F*_1_, ..., *F_n_*, we assert: Rep(R) SubClassOf: has-function some*F*_1 _and ... and has-function some*F_n _*and part-of some Rep(M).

• If a reaction *R *in a model *M *is annotated (using the BioModels.net biology qualifiers is, isVersionOf or hasVersion) with the process classes *P*_1_,..., *P_n_*, we assert: Rep(R) SubClassOf: has-function some (realized-by only*P*_1_) and ... and has-function some (realized-by only*P_n_*) and part-of some Rep(M).

• If a model entity *E *is annotated (using the BioModels.net biology qualifier isPartOf) with an entity *E*, we assert: E SubClassOf: part-of some Rep(E), where *Rep*(*E*) is the class that *E *would represent if it was annotated with the biology qualifier isVersionOf.

• If a model entity *E *is annotated (using the BioModels.net biology qualifier hasPart) with an entity *E*, we assert: E SubClassOf: has-part some Rep(E), where *Rep*(*E*) is the class that *E *would represent if it was annotated with the biology qualifier isVersionOf.

### Ontology, reasoning and model processing infrastructure

To perform the formalization of models, we used the Gene Ontology, the ChEBI Ontology, the Celltype Ontology and the PATO Ontology, all of which are freely available from http://obofoundry.org. Our software is implemented in Groovy and relies on the Manchester OWL API [91], the Pellet OWL reasoner [41], the Jena RDF library [92] and the libSBML [8].

## Authors' contributions

RH conceived of, designed, implemented and evaluated the method. MD, GVG and SW contributed to the evaluation, DLC, MD, GVG, JHG, and SW contributed to the design and discussion of the method. GVG and RH created the initial draft of the manuscript. All authors critically revised the manuscript. BdB, DLC and GVG supervised the project. All authors read and approved the final manuscript.
